# RETRACTION: Comment on: ‘Sex differences in neural networks recruited by frontloaded binge alcohol drinking’

**DOI:** 10.1111/adb.70068

**Published:** 2025-07-14

**RETRACTION**: J. Xu, H. Zhao, and Y. Wang, “Comment on: ‘Sex differences in neural networks recruited by frontloaded binge alcohol drinking’,” *Addiction Biology* 29, no. 10 (2024): e70002, https://doi.org/10.1111/adb.70002.

The above article, published online on 15 October 2024 in Wiley Online Library (wileyonlinelibrary.com), has been retracted by agreement between the authors; the journal Editor‐in‐Chief, Rainer Spanagel; the Society for the Study of Addiction; and John Wiley & Sons Ltd.

The retraction of this article, a Letter to the Editor, has been agreed by all parties due to concerns raised by a third party that it contains major scientific flaws. These flaws, which were confirmed during post‐publication review, were not identified during the original evaluation process and make the letter irrelevant to the published article on which it comments.

